# Osteocytes as main responders to low-intensity pulsed ultrasound treatment during fracture healing

**DOI:** 10.1038/s41598-021-89672-9

**Published:** 2021-05-13

**Authors:** Tatsuya Shimizu, Naomasa Fujita, Kiyomi Tsuji-Tamura, Yoshimasa Kitagawa, Toshiaki Fujisawa, Masato Tamura, Mari Sato

**Affiliations:** 1 Oral Biochemistry and Molecular Biology, Graduate School of Dental Medicine, Hokkaido University, Kita 13, Nishi 7, Kita-ku, Sapporo, 060-8586 Japan; 2 Oral Diagnosis and Medicine, Graduate School of Dental Medicine, Hokkaido University, Sapporo, Japan; 3 Dental Anesthesiology, Graduate School of Dental Medicine, Hokkaido University, Sapporo, Japan

**Keywords:** Experimental models of disease, Mechanisms of disease

## Abstract

Ultrasound stimulation is a type of mechanical stress, and low-intensity pulsed ultrasound (LIPUS) devices have been used clinically to promote fracture healing. However, it remains unclear which skeletal cells, in particular osteocytes or osteoblasts, primarily respond to LIPUS stimulation and how they contribute to fracture healing. To examine this, we utilized medaka, whose bone lacks osteocytes, and zebrafish, whose bone has osteocytes, as in vivo models. Fracture healing was accelerated by ultrasound stimulation in zebrafish, but not in medaka. To examine the molecular events induced by LIPUS stimulation in osteocytes, we performed RNA sequencing of a murine osteocytic cell line exposed to LIPUS. 179 genes reacted to LIPUS stimulation, and functional cluster analysis identified among them several molecular signatures related to immunity, secretion, and transcription. Notably, most of the isolated transcription-related genes were also modulated by LIPUS in vivo in zebrafish. However, expression levels of early growth response protein 1 and 2 *(Egr1*, *2)*, *JunB*, forkhead box Q1 *(FoxQ1)*, and nuclear factor of activated T cells c1 *(NFATc1)* were not altered by LIPUS in medaka, suggesting that these genes are key transcriptional regulators of LIPUS-dependent fracture healing via osteocytes. We therefore show that bone-embedded osteocytes are necessary for LIPUS-induced promotion of fracture healing via transcriptional control of target genes, which presumably activates neighboring cells involved in fracture healing processes.

## Introduction

Fracture healing is a complex, well-orchestrated, regenerative process involving various tissues and cell types. The repair process is divided into four main stages: inflammation, soft callus formation, hard callus formation, and remodeling^1, 2^. The injury initiates an inflammatory response with white blood cells accompanied by the secretion of inflammatory cytokines. This response induces coagulation of the hematoma around the fracture site and is a template for callus formation. A cartilaginous callus is formed by a collagen matrix produced by specific mesenchymal stem cells derived from the surrounding soft tissues and bone marrow. Within the callus, endochondral formation and endochondral ossification occur. The primary soft callus is resorbed by osteoclasts and replaced with a hard callus by osteoblasts. The hard callus is subsequently resorbed by osteoclasts for remodeling into the bone’s original cortical structure, characterized by coupled cycles of osteoblast and osteoclast activity. During this period, marrow space is re-established, vascular remodeling takes place, and, finally, new bone is generated. Thus, fracture healing requires the appropriate interaction of various tissues and cell types at each step^3^.

Mechanical stimulation by treatment with low-intensity pulsed ultrasound (LIPUS) is an established therapy for bone fracture treatment, and the US Food and Drug Administration approved EXOGEN (a LIPUS system) in the 1990s for the accelerated healing of certain fresh fractures^4^. Because mechanical loading, generally supplied by gravity and exercise, is effective at inducing bone remodeling and maintaining bone mass due to the regulating biological functions of bone cells^5, 6^, it seems reasonable to assume that LIPUS stimulation promotes fracture healing through control of bone cells. Both bone-forming osteoblasts and mechanosensitive osteocytes are capable of responding to LIPUS, and the synergistic action of these cells is likely important for the efficacy of LIPUS treatment^7^. It has been reported that osteoblasts are sensitive to LIPUS and contribute to fracture healing through bone formation and inflammatory regulation^8^. On the other hand, although they are less well studied than osteoblasts, osteocytes are also known to respond to LIPUS and have an important role in the biological function of fracture healing^9, 10^.

Osteocytes comprise over 90% of all bone cells that reside in the bone matrix, and they form the lacunar-canalicular network found throughout bone tissue^11^. Osteocytes sense mechanical stimuli through their unique morphologies, generate biological signals that affect osteoblasts and osteoclasts via osteocytic projections that reach the bone surface, and organize bone formation and resorption^12^. LIPUS stimulation (providing an acoustic radiation force) of bone expands surface waves and induces fluid flow in the osteocyte lacunar–canalicular network. Li et al. reported that LIPUS-stimulated murine long bone osteocyte Y4 (MLO-Y4) cells secreted prostaglandin E2 (PGE2) and NO into culture media, and the differentiation of osteoblastic cells cultured in this media were changed significantly^9^. Another method to apply mechanical stress, fluid-flow-induced shear stress, increases PGE2 release and cyclooxygenase 2 (*Cox2*) mRNA expression in osteocytic MLO-Y4 cells, and PGE2 is involved in the upregulation of connexin 43-based gap junctions in MLO-Y4 cells^13^. Furthermore, fluid-flow-induced shear stress enhances the mRNA and protein expression of anabolic and metabolic factors such as Insulin-like growth factor 1 (IGF1), mechano growth factor (MGF), vascular endothelial growth factor (VEGF), and hepatocyte growth factor (HGF) in MLO-Y4 cells^14^. Considering osteocyte-specific mechanosensing role, osteocytes rather than osteoblasts would be important to LIPUS effect. However, it has not been confirmed whether osteocytes are necessary and valuable for LIPUS-mediated fracture healing with an in vivo study.

It is difficult to conduct long-term targeted ablation of osteocytes in mammalian models due to the origin of osteocytes. Since osteocytes are differentiated osteoblasts, if they are removed from bone tissue, the remaining osteoblasts will become new embedded osteocytes within a few months. In this study, to address this issue, we used two common laboratory fish species: medaka (*Oryzias latipes*) and zebrafish (*Danio rerio*). Fish bone has the same components as mammalian bone: minerals, water, collagen, cells, and proteins^15^. As in mammals, bone-modelling processes are carried out by osteoblasts and osteoclasts in all teleost fishes^16^. However, most advanced fishes called neoteleosts including medaka, completely lack bone-embedded osteocytes^17^. Medaka and zebrafish have previously been used as fracture healing models, and bone fractures in the caudal bony fin rays of both species are repaired via the four healing processes of inflammation, soft callus formation, hard callus formation, and remodeling^18, 19^. These reports suggest that osteoblasts, and not osteocytes, are essential for the autonomous osteogenic functions induced by mechanical loading and fracture healing. However, we mammalian have osteocytic bone probably because of its functional evolutionary advantage, since osteocytes support and control osteogenesis by balancing the functions of osteoblasts and osteoclasts^20^. Furthermore, osteocytes are involved in the physiological function not only of osteogenesis, but also of lymphogenesis participating in immunity^21^.

In this report, we show that osteocyte-embedded zebrafish bone is more receptive to the beneficial effects of LIPUS during fracture healing. In addition, global gene expression analysis suggests that LIPUS-stimulated osteocytes may indirectly modify the function of osteogenic and inflammatory cells to accelerate fracture healing via the target genes of transcription factors regulated by LIPUS.

## Results

### LIPUS promotes fracture healing in zebrafish but not in medaka

**Figure 1:**
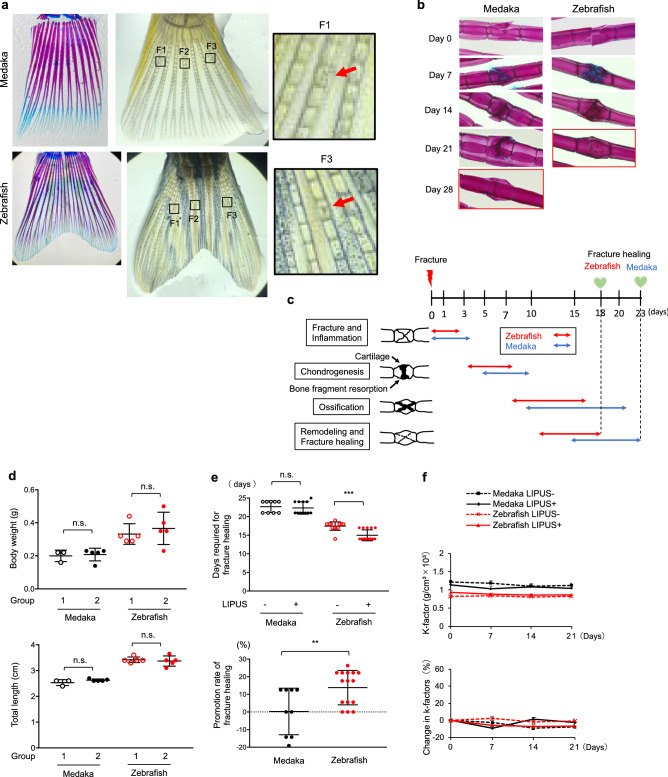
The effect of LIPUS treatment on fracture healing in medaka and zebrafish (**a**) Left: Tail bones of medaka and zebrafish stained by Alcian blue and Alizarin red. Middle: In tail bones of zebrafish and medaka, three fractures (F1, F2 and F3) per fish were induced with a scalpel under a microscope. Right: High magnification images of the boxed regions (medaka F1, zebrafish F3) in middle panels. Original magnification 2× (left and middle panel), 10× (right panel). (**b**) Stained images of the fracture healing process in medaka and zebrafish without LIPUS stimulation. The fracture site was stained with Alcian blue and Alizarin red. Original magnification: 10×. (**c**) Schematic representation of sequence of fracture healing processes in medaka and zebrafish. Inflammation stage, soft callus formation stage (chondrogenesis), hard callus formation stage (ossification), and remodeling stage are indicated by arrows (Red arrows: zebrafish, Blue arrows: Medaka). (**d**) The body weight (upper panel) and total length (lower panel) of experimental group of medaka and zebrafish before the LIPUS application (n = 3–5). (**e**) Upper: Days required for complete fracture healing in LIPUS-treated and untreated medaka and zebrafish. Lower: Promotion rate of fracture healing induced by LIPUS stimulation in medaka and zebrafish (n = 3–5 fishes × 3 fractures, representative data). The time point that the fracture was completely healed was measured in a blinded manner. This experiment was repeated three times with similar results. (**f**) Upper: The body weight and length of the fish were measured every week, and the condition factor (K-factor; g/cm^2^ × 10^3^) was calculated at the indicated points. Lower: Percent change in condition factor (n = 3–5). Data are presented as mean ± SEM. *p* Values ​​were determined using Student’s *t*-test. * *p* < 0.05, ** *p* < 0.01, *** *p* < 0.001.The photographs in (**a**) and (**b**) were taken by T. S.

To examine whether osteocytes are necessary for LIPUS to affect fracture healing, we compared fracture healing rates in medaka and zebrafish. Medaka have acellular bone lacking osteocytes, whereas zebrafish have osteocyte-containing bone, but apart from this both types of bone are extremely similar in structure, mechanical properties, and mineral density^15^. The fin rays contain segmented dermal bones and we fractured some of these bones using a scalpel in three places in each fish (Fig. 1a).

Alizarin red and Alcian blue staining was performed to observe the fracture healing processes in both species (Fig. 1b). After the fracture, blood clot forms hematoma which induce inflammatory response and be a template for callus formation. About 1 week, the fractured region formed an Alcian blue-positive callus and was bridged. About 2 weeks, the callus was gradually replaced by an Alizarin red-positive hard callus. About 3 weeks, the Alizarin red-positive calluses were then remodeled into a smooth surface. Since the surface was still not back to normal after a few months, at this point we considered the bone fracture have completely healed. The time frame of each phase of fracture healing, inflammation, chondrogenesis, ossification and remodeling, in medaka and zebrafish are depicted in Fig. 1c. The medaka bone took approximately seven days longer to heal completely than the zebrafish bone (Fig. 1b,c). There was no difference in the process and the period of fracture healing between male and female in both species.

Next, we examined the effect of LIPUS on accelerating bone fracture healing in anosteocytic and osteocytic bone with medaka and zebrafish. In fish fin rays, bone segments in the distal fin were Alcian blue positive unmineralized cartilage (Fig. 1a) because fish fins grow by sequentially adding new segments of bone to the distal end of fin ray^22^. We selected Alizarin red positive mineralized bone located in proximal part of fin ray to induce bone fracture and three fractures per fish were induced (Fig. 1a). Before the LIPUS treatment, medaka and zebrafish were divided into two groups of each and we confirmed that there was no difference in the total length and body weight between two groups (Fig. 1d). Then, the fish divided into two groups were stimulated or unstimulated with LIPUS for 20 min at 30 mW/cm^2^ once a day until the fracture was completely healed. Three fractures per fish scored separately because there are about 1–3 days differences in healing time within a fish. The LIPUS treatment accelerated fracture healing in the zebrafish but not in the medaka (Fig. 1e). The condition factor (K-factor: weight [g]/(length [cm]^3^) × 1000) that is used as an indicator of the physiological state of fish dependent on adequate management (feeding density and climate) was not affected by the LIPUS treatment in either species (Fig. 1f). The LIPUS treatment provided an advantage during fracture healing, probably through direct effects on the skeletal tissue rather than causing systemic changes in physiological condition. Because the effect of LIPUS on fracture healing is apparent in zebrafish bone, which contains osteocytes, but not in medaka bone, which does not, osteocytes appear to be important for LIPUS-mediated fracture healing.

### Global transcriptome analysis in LIPUS-stimulated osteocytes

**Figure 2:**
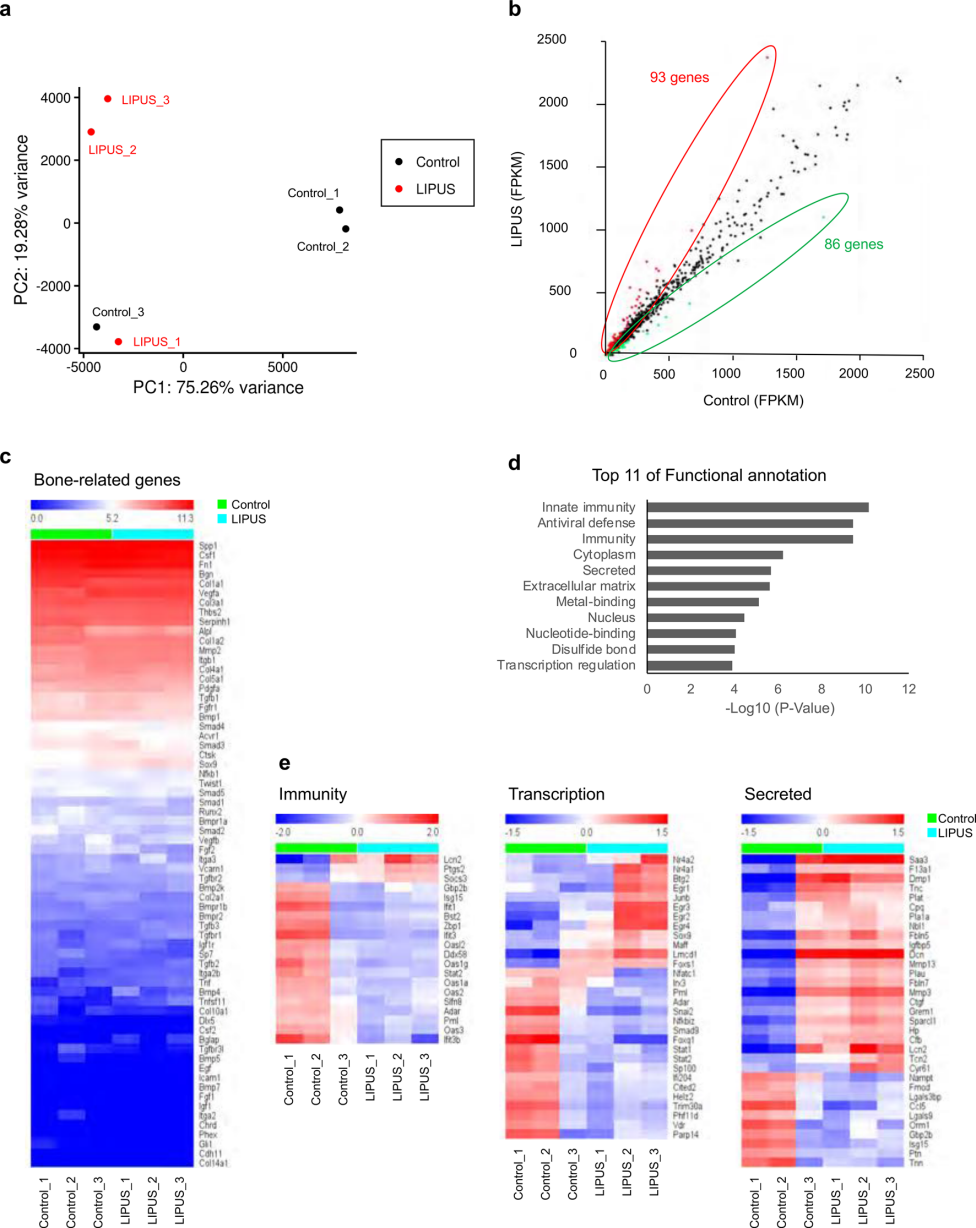
RNA-seq analysis of LIPUS-stimulated MLO-Y4 cells. (**a**) Principal Component Analysis (PCA) plot showing the variability between the biological replicates of control and LIPUS-treated MLO-Y4 cells. (**b**) Comparison of gene expression levels by scatter plot. In total, 179 genes were identified as significantly affected, of which 93 were upregulated (red outline) and 86 were downregulated (green outline) by LIPUS. (**c**) Heat map showing the change in expression of bone-related genes in LIPUS-treated and untreated MLO-Y4 cells. (**d**) Using the 179 affected genes, affected annotated term categories were identified. Log_10_ (*p* value) > 1.3 (*p* value < 0.05) was considered significantly different. (**e**) Heat map showing the change in expression of genes involved in immunity (left), transcription (center), and secretion (right) in LIPUS-treated and untreated MLO-Y4 cells (n = 3 biological independent samples per group). (**a**) was created by N. F. using PCAGO (https://pcago.bioinf.uni-jena.de). (**b**), (**c**) and (**e**) were created by N. F. using R programming language (v3.6.3; http://www.r-project.org). (**d**) was created by N. F. using DAVID (v6.8; https://david.ncifcrf.gov).

Osteocytes therefore responded to LIPUS stimulation and contributed to the accelerated fracture healing. In order to understand the molecular events in osteocytes triggered by LIPUS, we performed RNA sequencing (RNA-seq) analysis of MLO-Y4 oateocyte-like cells exposed to LIPUS and of controls. Cells were stimulated for 20 min with LIPUS and subsequently placed in a CO_2_ incubator for 30 min and RNA was then extracted. The experiment was repeated independently three times and the transcriptomes were determined by using three independent RNA samples. Principal component analysis (PCA) was performed to investigate the variability between the biological triplicates of LIPUS treated and non-treated samples (Fig. 2a). The first component (PC1) that separates two of control samples from LIPUS-treated group represents more than 75% of the variability. However, one of the control biological replicates differ from the other two samples. Since LIPUS treated and non-treated (control) cells were generated in one cell culture plate, leakage of LIPUS stimulation into control cells may lead to the variation among control group. Of 16,476 genes identified, 179 were significantly affected by LIPUS stimulation (Fig. 2b). Of these 179 genes, 93 were upregulated and 86 were downregulated by LIPUS stimulation (Fig. 2b). Since the expression levels of most of the bone-related genes did not change (Fig. 2c), LIPUS-stimulated osteocytes may participate in fracture healing not through their osteogenic function.

To clarify the biological meaning of these 179 genes, functional annotation analysis was performed using the functional annotation tool DAVID and Uniprot keywords were selected as the database. Top-ranked major annotated term categories included Immunity, Antiviral defense, Cytoplasm, Secreted, Extracellular matrix, and Transcription (Fig. 2d). Further, these genes were clustered into nine groups, and enrichment scores greater than 1.3 (which corresponded to the negative log of *q*-values < 0.05) were considered significant, resulting in seven clusters that were significantly enriched (Table 1). Similarly to the annotation categories, Immunity, Transcription, and Secreted (bolded in Table 1) genes were in the top four result clusters. It seems reasonable that LIPUS modulates these functions in osteocytes, because in fracture healing an inflammatory immune response is necessary and osteocyte-derived secreted factors are likely to play a role in the recruitment of mesenchymal stem cells (MSCs) and blood cells to form a callus and induce vascularization.

**Table 1:** List of enriched annotation clusters of the 179 LIPUS-responsive genes.

Annotation Cluster	Name	Gene Count	P_Value	Gene name included in									
Cluster A Enrichment Score 7.44	Innate immunity	17	7.10E−11	**Immunity**									
	Antiviral defense	12	3.50E−10	*Oas1a*	*Oas2*	*Oas3*	*Oasl2*	*Ddx58*	*Zbp1*	Adar* (NM_001146296.1)	Bst2* (NM_198095.3)	C1rb* (NM_001113356.1)	C1s2* (NM_173864.2)
	**Immunity**	**20**	3.60E−10	Cfb* (NM_008198.2)	*Gbp2b*	Hp* (NM_017370.2)	*Irgm1*	*Iigp1*	*Ifit1*	*Ifit3*	*Lgals9*	*Lcn2*	*Pml*
	RNA-binding	8	1.90E−01										
Cluster B Enrichment Score 3.88	Nucleus	59	3.40E−05	**Transcription**									
	Transcription regulation	30	1.30E−04	Btg2* (NM_007570.2)	Cited2* (NM_010828.3)	Irx3* (NM_008393.3)	Lmcd1* (NM_144799.2)	*Phf11d*	Smad9* (NM_019483.5)	Sox9* (NM_011448.4)	Adar* (NM_001146296.1)	Egr1* (NM_007913.5)	Egr2* (NM_010118.3)
	**Transcription**	**30**	2.30E−04	Egr3* (NM_018781.4)	*Egr4*	Foxq1* (NM_008239.4)	*Foxs1*	Helz2* (NM_183162.2)	*Ifi204*	Junb* (NM_008416.3)	*Sp100*	Nfatc1* (NM_001164111.1)	Nfkbiz* (NM_001159394.1)
	DNA-binding	27	2.80E−04	Nr4a1* (NM_010444.2)	*Nr4a2*	*Parp14*	*Pml*	*Stat1*	*Stat2*	Snai2* (NM_011415.3)	*Trim30a*	*Maff*	*Vdr*
Cluster C Enrichment Score 3.3	Metal-binding	50	7.70E−06										
	Zinc	30	1.60E−03										
	Zinc-finger	22	1.00E−02										
Cluster D Enrichment Score 3.21	**Secreted**	**33**	2.00E−06	**Secreted**									
	Disulfide bond	44	1.00E−04	Isg15* (NM_015783.3)	Sparcl1* (NM_010097.4)	*Cpg*	Ccl5* (NM_013653.3)	F13a1* (NM_028784.3)	Cfb* (NM_008198.2)	Ctgf/Ccn2* (NM_010217.2)	Cyr61/Ccn1* (NM_010516.2)	Dcn* (NM_001190451.2)	*Dmp1*
	Signal	49	1.20E−02	*Fmod*	Fbln5* (NM_011812.4)	Fbln7* (NM_024237.4)	Grem1* (NM_011824.4)	*Gbp2b*	Hp* (NM_017370.2)	*Igfbp5*	*Lgals9*	*Lgals3bp*	*Lcn2*
	Glycoprotein	39	5.80E−02	Mmp13* (NM_008607.2)	*Mmp3*	*Nbl1*	*Nampt*	*Orm1*	Pla1a* (NM_134102.4)	*Plat*	*Plau*	*Ptn*	Saa3* (NM_011315.3)
				Tnc* (NM_001369211.1)	*Tnn*	*Tcn2*							
Cluster E Enrichment Score 1.48	ATP-binding	19	1.90E−02										
	Magnesium	10	2.10E−02										
	Transferase	19	9.20E−02										
Cluster F Enrichment Score 1.32	Lipid biosynthesis	6	8.30E−03										
	Cholesterol biosynthesis	3	1.00E−02										
	Sterol biosynthesis	3	1.70E−02										
	Steroid biosynthesis	3	3.50E−02										
	Fatty acid biosynthesis	3	6.60E−02										
	Cholesterol metabolism	3	6.80E−02										
	Sterol metabolism	3	8.40E−02										
	Lipid metabolism	7	1.10E−01										
	Steroid metabolism	3	1.10E−01										
	Fatty acid metabolism	3	2.50E−01										
Cluster G Enrichment Score 1.31	Microsome	6	3.70E−03										
	Oxidoreductase	10	6.20E−02										
	Heme	4	1.50E−01										
	Iron	6	1.70E−01										

These transcription factors may also be important in regulating related gene expression in all fracture healing processes, including the inflammatory and bone remodeling phase. We investigated individual gene expression in three clusters: Immunity, Secreted, and Transcription. 23 of 33 genes genes belonging to the Secreted category were upregulated by LIPUS, whereas 17 of 20 Immunity genes tended to be downregulated (Fig. 2e). In the Transcription category, there were almost equal numbers of up- (12) and downregulated (18) genes after LIPUS stimulation (Fig. 2e). These results show that osteocytes respond to LIPUS via 179 genes that are involved in the inflammatory response, transcriptional regulation, and protein secretion, all of which affect the fracture healing rate.

### Gene expression altered by LIPUS in zebrafish fin rays

Next, to examine whether genes in the categories Immunity, Transcription, and Secreted are also modulated by LIPUS in vivo gene expression levels of each cluster category were measured in zebrafish fins. Bone fractures were induced in caudal fins and stimulated with LIPUS for 20 min once a day. On days 1 and 7, RNA was isolated from the fin rays 30 min after stimulation. To perform qPCR with these samples, we isolated the appropriate genes using the steps described below for the Immunity category in cluster A, which included 20 genes (Table 1). From among these genes, we first selected the 14 that had the smallest *q*-value (0.00388). Next, we eliminated genes that do not have zebrafish orthologs or for which it was not possible to design primer sets for qPCR. Finally, six genes were left (labeled with asterisks in Table 1) and we measured the changes in their expression using qPCR. Similarly, 17 genes were selected from the Transcription category in cluster B and 16 from the Secreted category in cluster D for qPCR (labeled with asterisks in Table 1).

**Figure 3:**
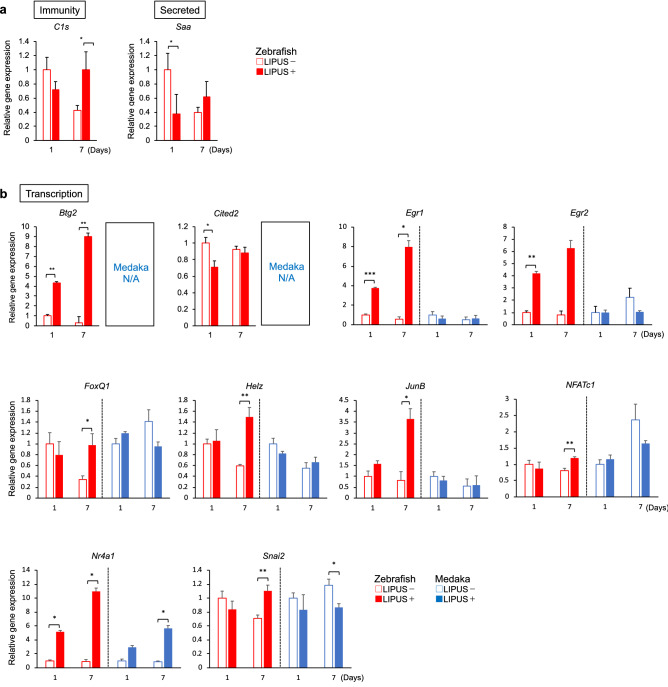
Measurement of candidate gene expression in LIPUS-treated zebrafish and medaka. Zebrafish and medaka tail bones were fractured and stimulated with LIPUS for 20 min every day. On days 1 and 7 after the fracture, RNA was extracted from the fin rays. (**a**) Immunity-, Secretion-, and (**b**) Transcription-related genes were measured (n = 3). For medaka, *Btg2* and *Cited2* couldn’t design qPCR primers because reference sequences were not available (N/A) in the public database. Values presented are the mean ± SEM and the significance of differences was determined using Student’s *t*-test. * *p* < 0.05; ** *p* < 0.01.

Most of the genes in the Immunity and Secreted categories were not affected by LIPUS stimulation of the zebrafish fins. In the Immunity category, only one out of six genes (16.6%), *C1s2* (Complement component 1, s subcomponent 2), was upregulated, and in the Secreted category the expression of only one out of 16 genes (1.5%), *Saa3* (Serum amyloid A-3 protein), was affected (Fig. 3a). However, the expression of 10 out of the 17 genes (58.8%) in the Transcription category was significantly altered (Fig. 3b). Reactivity to LIPUS of the Transcription-classified genes was approximately parallel in MLO-Y4 cells and zebrafish fin rays. Therefore, we focused on Transcription-categorized genes for target gene analysis.

### Target gene analysis of transcription factors modulated by LIPUS

To identify the target genes regulated by the 10 LIPUS-responding transcription factor genes, we utilized the online ChIP-seq database ChIP-Atlas and obtained the binding scores for *Egr1*, *Egr2*, nuclear receptor 4a1 *(Nr4a1)*, *JunB*, and *NFATc1* in the promoter regions of target genes. Unfortunately, information regarding *Btg2*, *Snai2*, *Cited2*, *FoxQ1*, and *Helz2* was not available in this database (labeled N/A in Table 2). The top 10 potential target genes with the highest average scores were selected and their expression levels in osteocytic MLO-Y4 cells were evaluated using RNA-seq data (column 4 in Table 2). RNA-seq data showed that all target genes were not changed by LIPUS (column 4–6 in Table 2), probably because there is a time-lag for target genes to be initiated transcription by these transcription genes.

**Table 2:** List of genes targeted by transcription factor genes.

Transcription genes	Target genes	Average score	Expression level in MLO-Y4 (FPKM value)		Significant	Function	Refs.
			LIPUS −	LIPUS +			
*Egr1*	*Greb1*	1109.833333	1.53125	1.21937	no		
	*Olfr806*	790.5	n.d	n.d	no		
	*Abhd6*	774.166667	1.07994	0.848552	no		
	*Wwox*	725.666667	6.25733	5.4122	no		
	*Aadacl2fm1*	613.666667	n.d	n.d	no		
	*Nkx2-6*	581.5	n.d	n.d	no		
	*Setd4*	549.333333	0.960806	0.709573	no		
	***Cbr1***	549.333333	**31.0524**	28.1055	no	Carbonyl reductases, Catalyst for the reduction of endogenious prostaglandine and steroids	^24^
	*Cldn34d*	526.666667	n.d	n.d	no		
	*Tecpr1*	434.166667	2.83544	3.10746	no		
*Egr2*	***Dynlt1b***	756	**34.0942**	34.2265	no	Activators of G-protein signaling	PMID: 11358340
	*Lcmt1*	749.909091	8.62011	10.0269	no		
	***Arhgap17***	749.909091	**15.6857**	17.1241	no	Rho GTPase activating proteins, Maintenance of tight junctions	PMID: 16678097
	***Ncapg2***	746.181818	**27.2157**	32.8379	no	A component of the chromosome condension II complex, which is critical for mitosis	PMID: 31176678
	*Mtrf1l*	740.818182	3.14548	5.9366	no		
	*Sdhaf3*	712.454545	0.920439	1.18144	no		
	*D10Wsu102e*	690.090909	1.87224	1.91581	no		
	*Cenpu*	677.818182	15.7252	21.934	no		
	*Cplane1*	676.272727	6.84822	5.60805	no		
	*Cmtm6*	647.636364	7.80277	9.01462	no		
*Nr4a1*	***Adssl1***	811.428571	**24.6413**	38.4503	no	Adenylosuccinate synthetase, which catalyzes AMP synthesis	PMID: 15786719
	***Mcl1***	809.285714	**126.123**	156.683	no	Anti-apoptotic protein, pro-survival protein	PMID: 20023629
	*Txk*	800.285714	n.d	n.d	no		
	*Tmem179*	767.428571	1.11521	4.11481	no		
	*Cpm*	706.142857	3.52267	3.59489	no		
	*Greb1*	679.428571	1.53125	1.21937	no		
	*Olfr266*	655	n.d	n.d	no		
	*Rapgef2*	630.285714	5.72616	5.78346	no		
	***Cst3***	607.857143	**454.443**	593.939	no	Cysteine proteinase inhibitor, An inhibitor of bone resorption	^25^
	***Brox***	606.285714	**15.5085**	15.461	no	Apoptosis-linked gene, Controlling of cell death through the regulation of the endolysosomal system	PMID: 16407552
*JunB*	*Cldn34d*	895.351351	n.d	n.d	no		
	***Brox***	763.783784	**15.5085**	15.461	no	Apoptosis-linked gene, Controlling of cell death through the regulation of the endolysosomal system	PMID: 16407552
	***Aida***	763.783784	**67.1444**	55.861	no	Axin-interacting protein, Blocking Axin/JNK signaling	
	*Cdc42bpg*	643.567568	5.53688	6.65211	no		
	*Mefv*	604.567568	n.d	n.d	no		
	*Ank1*	598.972973	7.66976	6.28425	no		
	*Prss56*	598.027027	0	0.216514	no		
	*Chrnd*	598.027027	n.d	n.d	no		
	*Cass4*	595.810811	0.0982373	0.0633786	no		
	***Htra3***	574.432432	**10.6126**	13.0335	no	Serine protease, An inhibitor of TGF-b signaling	^23^
*Nfatc1*	*Greb1*	1166	1.53125	1.21937	no		
	*Olfr855*	844.333333	n.d	n.d	no		
	*Olfr854*	844.333333	n.d	n.d	no		
	*Abhd6*	796.333333	1.07994	0.848552	no		
	*Nkx2-6*	765.333333	n.d	n.d	no		
	*Wwox*	748.333333	6.25733	5.4122	no		
	*Olfr806*	723	n.d	n.d	no		
	*Acacb*	657.666667	0.787511	0.493981	no		
	*Prl3c1*	591.666667	n.d	n.d	no		
	*Setd4*	562.333333	0.960806	0.709573	no		
*Btg2*	N/A						
*Snai2*							
*Cited2*							
*FoxQ1*							
*Helz*							

Eleven genes which abound with high expression levels in MLO-Y4 cells (FPKM value > 10; bolded in Table 2) were selected and their functions were investigated. These genes participate in energy metabolism (*Adssl1*), cell survival (*Ncapg2*, *Mcl1*, and *Brox*), and cell–cell contact (*Arhgap17*), and involve several signaling pathways (*Cbr1*, *Dynlt1b*, *Aida*, and *Htra3*) (Table 2). The TGF-β and Cox2/PGE2 pathways, which are modulated by *Htra3* and *Cbr1* factors^23, 24^, are especially interesting because they are induced and secreted in the early stages of fracture healing (inflammation phase) as proinflammatory molecules^3^. *Cst3*, which is the most abundantly expressed gene in MLO-Y4 cells, encodes a secreted factor and promotes the differentiation of osteoblasts associated with BMP signaling^25^.

Previous reports were referred to for other transcription factor genes (*Btg2*, *Snai2*, *Cited2*, *FoxQ1*, and *Helz2*) because their target genes were not available in the ChIP-Atlas database. *Btg2* can code for transcriptional coactivators and allows interaction with and modulation of various nuclear receptors, such as all-trans retinoic acid receptors, estrogen receptors, and androgen receptors^26^. It has been reported that estrogen receptors in osteocytes are important for bone formation^27^. *Snai2* factors can act as transcriptional repressors and regulate cell apoptosis, migration, and detachment from and attachment to the extracellular matrix^28^. *Cited2* encodes a CBP/p300-binding transcription coactivator and enhances TGF-β-mediated transcription^29^. Interestingly, a previous study using a rat fracture model reported that a *Cited2* factor was a negative regulator of fracture healing associated with an increase in matrix metalloprotease^30^. LIPUS stimulation reduced *Cited2* expression in the fractured fin rays (Fig. 3b), which is consistent with the positive effect of LIPUS on fracture healing. *FoxQ1* encodes a member of the forkhead transcription factor family and regulates cell proliferation via TGF-β and Wnt signaling^31^. *Helz2* encodes transcriptional coactivator helicase with zinc finger 2, a coregulator of peroxisome proliferator-activated receptor gamma (PPARγ)^32^. Since PPARγ in osteocytes is important for the coupling of bone formation and resorption^33^, the upregulation of *Helz2* by LIPUS treatment (Fig. 3b) may control fracture healing processes such as bone remodeling from osteoblasts and osteoclasts.

Taken together, these results show that the 10 transcription genes shown in Table 3 were modulated by LIPUS stimulation not only in vitro but also in vivo. Further, their target genes may be involved in the promotion of fracture healing, probably through activation of the inflammatory response and osteogenic cell differentiation.

**Table 3:** Primer sequences for qRT-PCR.

Gene (Danio rerio)	NCBI Entrez ID	Ref-Seq	Primer sequence (Forward)	Primer sequence (Reverse)
*C1s.1*	*793529*	NR_027750.1	GACCTGTGACGCCAACATCTA	GGATAACCGGACTCCACTGTC
*Saa*	*449557*	NM_001005599.2	CAAGTATTTCCATGCACGCGG	GCAGCATCTGAATTGCCTCTG
*Btg2*	*30079*	NM_130922.2	CATTCTGATCTTTGCCGGACG	GGAACCAATGGTGCTGGTAGT
*Cited2*	*450024*	NM_001006045.1	GGAGAGCATACGCTTCTTGTTG	CGACCATGGTTCATTGCCATC
*Egr1*	*30498*	NM_131248	TTCTCAACGCCACAGCACCTGAAGG	GGTCTGATCTGACAGAGGTTTCTCC
*Egr2a isoform 1*	*368241*	NM_001328404.2	GTCTATGGTCTGGATGAGATTCCC	AGATCAAGGTCCCGCTTTTCC
*FoxQ1b*	*405843*	NM_212907.1	TGGAGGTTTTCTCTGCGAGTC	TAAGGTGGTTTGGGTCTACGC
*Helz2a*	*562461*	XM_003198847.4	GGACTGCCTCGTTTCACTGTA	CCAATAGGCTGTCCTTGGTGT
*JunBa*	*407086*	NM_213556.3	GACCCTCCGCTCCGAAAT	TGATGCTCCGACCGTACAAATA
*JunBb*	*336038*	NM_212750.1	TCCCACATACAGCAGAGCCA	GCGTTCCTGCGAGTCCAT
*NFATc1*	*568315*	NM_001045159.1	CAAGCATGAAATCCGCAGAGG	CCGGATGTTTGGAAGTAGCCT
*Nr4a1*	*431720*	NM_001002173.1	CCTCTCTCGTTACTGCCCATATC	CCTGATCACATCCATTGACCCTG
*Snai2*	*494038*	NM_001008581.1	CAGCATGCCTCGTTCATTCCT	CCGGGAGGGCTTTTAAGACATA
**Gene (Oryzias latipes)**		**Ref-Seq**	**Primer sequence (Forward)**	**Primer sequence (Reverse)**
*Egr1*	*100272163*	NM_001146145.1	CGTACGACCACCTTACTGGAG	GACCACTGAACAGACCCAAGA
*Egr2*	*101170128*	XM_004080784.4	GGCCACTACGACCAACTCAAT	GCTGGATAAGGGGAGTCGATG
*FoxQ1b*	*101164223*	XM_011474400.3	GGAAAGGGAAACCCTACACCC	TGAGGGAGAGGTTGTGTCTCA
*JunBb*	*101164062*	XM_004071833.4	CAACACTGAACGCCTATTGCC	GGGCTCCTCCTTCAAGGTAAC
*NFATc1transcript variant X1*	*101161493*	XM_004074292.4	TACAGGCAGGGAACACGTTTG	GAAACGTTCAGACTGTGGGTC
*Nr4a1transcript variant X1*	*101175402*	XM_004070882.4	CAATGCCTCCTGTCAGCACTA	GACACTTCTGGAAGCGACAGA
*Snai2*	*101173257*	XM_004079391.4	CCAAGATGCCACGCTCTTTTC	AGGCTACTGGTAGTCCACACT
*Helz*	*101160963*	XM_023960438.1	CTGACGACTAGATCCATGTACCG	TAGGGTCCAATGATGAGGATAGGG

### Measurement of candidate transcription genes affected by LIPUS in medaka fin rays

In zebrafish bone, which contains osteocytes, several transcription genes reacted to LIPUS under fracture-healing conditions. To assess whether osteocytes are important for LIPUS efficacy via these transcriptional factor genes, we performed another in vivo experiment with medaka, whose bone lacks osteocytes. Medaka fin rays with bone fractures were isolated after LIPUS treatment and RNA was extracted, as with the zebrafish samples. Of the ten transcription genes shown in Table 2, eight genes could measure by qPCR. Primer sets of other two genes were not be able to make because medaka reference sequences were not found in the public database.

Interestingly, *Egr1*, *Egr2*, *FoxQ1*, *Helz, JunB*, and *NFATc1* did not respond to LIPUS in the medaka (Fig. 3b); these are considered osteocyte-dependent genes that are induced by LIPUS treatment. The expression of other genes, however, such as *Snai2* and *Nr4a1*, was altered by LIPUS in the medaka, similarly to zebrafish (Fig. 3b). Since MLO-Y4 cells are osteocyte-like, in other words, late mature osteoblasts, MLO-Y4 cells seem to partially retain an osteoblastic signature^34^. Therefore, *Snai2* and *Nr4a1* may react to LIPUS not only via osteocytes but also via osteoblasts.

These in vivo results show that osteocytes contribute to LIPUS-promoted fracture repair via transcriptional regulation of *Egr1*, *Egr2*, *FoxQ1*, *Helz*, *JunB*, and *NFATc1*. This can induce cellular metabolism and survival mediated by the target genes or trigger various signaling pathways, such as the TGF-β- and Wnt-dependent pathways that are required for fracture healing.

## Discussion

**Figure 4:**
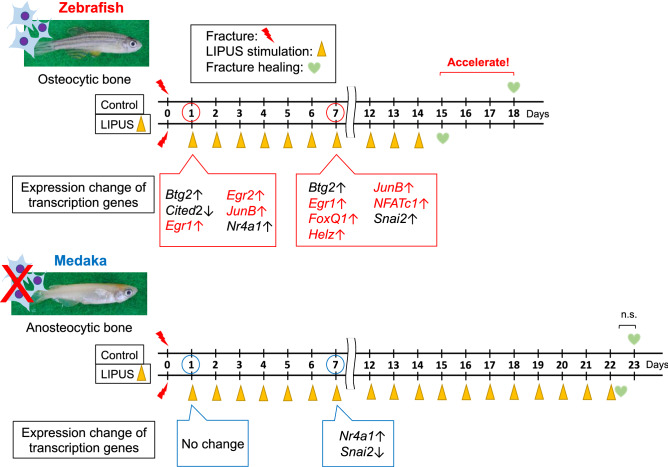
Schematic summary. Schematic representation of the LIPUS effect on the fracture healing and associated transcription factor genes in zebrafish which comprise osteocyte-containing bone and medaka which comprise osteocyte-lacking bone. The photographs of zebrafish and medaka were taken by M. S.

In this study, we demonstrated that zebrafish which have osteocytic bone, is more receptive to gain LIPUS-induced promotion of fracture healing than in medaka, which have anosteocytic bone. LIPUS stimulation altered the gene expression profile in osteocytes, and some of the transcription genes whose expression was affected appear to be important for exerting the role of osteocytes in modifying the function of osteogenic and inflammatory cells that are involved in the fracture healing process (Fig. 4).

Ample in vitro studies have shown that osteoblasts and chondrocytes rather than osteocytes, respond to LIPUS, and directly activating their functions and differentiation is important for LIPUS-dependent acceleration of fracture healing^35, 36^. Indeed, osteoblasts and chondrocytes directly generate new bone, and it has been proposed that they are specific key players in the endochondral ossification that is part of the fracture healing process. Whereas, osteocytes are terminally differentiated cells and do not exert a direct effect on osteogenesis, so how osteocytes contribute to the effect of LIPUS on fracture healing is unclear, despite their mechano-sensitivity. In vivo studies have demonstrated that LIPUS accelerates all stages of the fracture repair process (inflammation, bone formation, and bone remodeling), by enhancing the mineralization and the inflammatory response^1, 37, 38^. Naruse et al. found that *Cox2*-knockout mice failed to show LIPUS-accelerated fracture healing effects^39^, although Cox2 expression was observed in several cell types, including osteoblasts, osteocytes, chondrocytes, and inflammatory cells. To clarify the roles of osteoblasts and osteocytes in the response to LIPUS, in vivoexperiments with genetic animal models lacking each of these cell types would be useful. However, since osteocytes are derived from osteoblasts, it is difficult to conduct long-term targeted ablation of one of these cell types but not the other in animal models. Thus, in vivo studies still have not defined the main target cells contributing to the efficacy of LIPUS.

In this report, we utilized medaka as a natural osteocyte knock-out model to elucidate the effect of osteocytes in LIPUS-induced fracture repair. Zebrafish, which have osteocyte-rich bone, were utilized as a comparative model. Because the size, ecology, and swimming mode of both fish species are similar, they have previously been used in a comparative study to examine the role of osteocytes in bone modeling^2^. Comparisons of these species are partially suitable for bone research focusing on osteocytes, but the differences between them due to evolutionary distance should not be ignored. It is estimated from genomic comparisons that medaka and zebrafish separated from their last common ancestor approximately 110 million years ago^40, 41^. This evolutionary distance is reflected in their biological functioning. For example, regeneration of the heart following injury is observed in zebrafish and not in medaka^42^, and the response to the estrogen 17β-estradiol occurs faster in medaka than in zebrafish^43^. Although fracture healing processes seem to be similar in the two species, their biological differences could affect experiments on fracture healing with LIPUS stimulation. To obtaining the more precise findings by in vivo using fish, the present study, utilizing only medaka and zebrafish, is not sufficient. Other than zebrafish, goldfish (*Carassius auratus*), carp (*Cyprinus carpio*), catfish (*Mystus macropterus*), and salmon (*Salmo salar* and *Oncorhynchus*) also have osteocyte-containing bone. Like medaka, tilapia (*Oreochromis aureus*), fugu (*Takifugu oblongus*), and platyfish (*Xiphophorus maculatus*) do not have osteocytes in their skeletal tissue^44, 45^. Cohen et al. performed a comparative study with the common carp (with cellular bone) and tilapia (with acellular bone) to investigate the mechanical properties of the two types of bone tissue^46^. Further studies using other fish species are needed, to reveal more clearly the role of osteocytes in LIPUS efficacy.

We focused on *Egr1*, *Egr2*, *FoxQ1, Helz, JunB*, and *NFATc1*as osteocyte-specific LIPUS-sensitive genes. *Egr1* and *2*, and especially *Egr1*, have been reported to encode transcription factors that are induced by mechanical stimulation in cells such as endothelial cells^47^, tendon cells^48^, and myocytes^49^. In osteoblastic cells, 15 min of gravity loading induced an increase in the expression of *Egr1*^50^, and substrate stretching (Flexcell) also upregulated *Egr1* expression^51^. In osteocytic cells, *Egr1* expression was upregulated by a lack of the phosphate-regulating gene *PHEX*^52^ and activation of parathyroid hormone (PTH) signaling^53^. However, we found no studies indicating an interaction between mechanical stress, such as LIPUS treatment, and *Egr1* expression in osteocytes. Also, we could not find any research articles related to the role of *Helz* in bone cells including osteocytes and in mechanical stimuli. It has been reported that *JunB* expression is altered by mechanical stretching or loading in murine osteoblastic cells^54^ and in rat chondrocytes^55^, but not established in osteocytes. *FoxQ1* expression regulates the osteogenic differentiation of mouse bone MSCs^56^, but its role in osteocytes and osteoblasts is still unknown. *NFATc1* was highly expressed in the mouse parietal bones and MLO‐Y4 cells and nuclear translocation of *NFATc1* was induced by the compressive force in osteocytes embedded in murine parietal bones^57^. In the present study, we found several novel candidate transcription genes that are mechano-sensitive and contribute to the promotion of fracture healing by LIPUS, most likely in an osteocyte-dependent manner.

To predict the functional meaning of the LIPUS-regulated expression of *Egrs*, *FoxQ1*, *Helz*, *JunB* and *NFATc1*in osteocytes, we performed target gene analysis and focused on types of molecular signaling that may increase the potency of osteocytes in repairing bone fractures in cooperation with neighboring cells. The target genes of these transcription factors trigger TGF-β signaling, and PGE2 synthesis. A previous global gene expression analysis using exon arrays in rat bone following mechanical loading identified loading-induced genes and performed clustering analysis^58^. In that study, the forelimbs of rats were loaded for three minutes per day, and global gene expression changes were evaluated over a time course of four hours to 32 days. Early-response genes that were upregulated within 12 h included *JunB* but not *Egrs* or *FoxQ1*. Interestingly, many gene groups known to be essential for bone formation were identified within clusters related to matrix formation, Wnt/β‐catenin signaling, and TGF‐β signaling. This result was partially consistent with our RNA-seq analysis of MLO-Y4 cells treated with LIPUS.

Sclerostin (SOST) expressed in osteocytes is a key factor in regulating mechanical stimulation-induced bone change through TGF‐β signaling and PGE2 synthesis in mammalian. Osteocytes secrete the protein SOST, encoded by the *SOST* gene, which negatively regulates bone mass and osteoblast differentiation by inhibiting Wnt/β-catenin signaling^59^. It has been reported that SOST is induced by mechanical loading via a TGF-β-dependent mechanism^60^, and osteocyte-intrinsic TGF-β elevates the expression of *SOST* genes^61^. Indeed, in osteocyte-specific TGF-β receptor II-deficient mice, osteocyte-intrinsic TGF-β signaling maintained bone quality and fracture resistance through perilacunar–canalicular re-modeling, which involved the activity of osteocytes, osteoblasts, and osteoclasts^61^. Osteocytes also release PGE2, which may stimulate osteoblastic activity via control of the Wnt/β-catenin pathway^62^, probably because PGE2 represses SOST expression^63^. Interestingly, SOST and PGE2, which control osteoblastic activity, are modulated by mechanical loading in osteocytes and enhance bone formation and remodeling as a consequence of the mechanical response of osteocytes^64, 65^. In addition, TGF-β and Cox2/PGE2 are important molecules for causing inflammatory cells to commit to the early phases of fracture repair^3^. However, previous reports showed that SOST expression was not observed in osteocytes in both zebrafish and medaka^15^ and we confirmed that by qPCR with fin ray sample. MLO-Y4 cells is known to express a very low level of SOST and our RNA-seq data defined that. Therefore, although it is difficult that our results are extrapolated to mammalian system simply, our findings could be cue for investigation of SOST-independent alternative system for LIPUS-mediated fracture healing through TGF‐β signaling and PGE2 synthesis.

Despite the fact that the effect of “active” bone-forming osteoblasts and chondrocytes on LIPUS-induced fracture repair is well reported, the role of “static” osteocytes, which control bone generation indirectly, is still not fully understood. In addition, the synergistic activity of the many cell types triggered by LIPUS during fracture healing in situations mimicking physiological conditions remains to be elucidated. The present in vivo and in vitro studies show that osteocytes are LIPUS-sensitive cells, and the reactions they assist as a result of early-response transcription genes that affect bone-forming and inflammatory cells seem to be necessary for the promotion of fracture healing. However, further experiments are required to investigate whether the LIPUS-targeted signals and molecules in osteocytes do actually affect the functions of other collaborating cells, and to determine what kind of molecular and functional changes occur in other cells to influence biological or physiological activity during fracture repair. Because the molecular response of osteocytes to LIPUS can change in the long term as fracture healing progresses, it is also important to examine changes in the effect of LIPUS on the control of osteocytes over other cells and on the harmonization of the functions of various cells in each phase of fracture healing.

## Methods

### Ethics statement

There was no need of obtaining permissions for conducting experiments using fish in this study. In the current laws and guidelines of Japan relating to animal experiments of fish, experiments using fish are allowed without any ethical approvals from any authorities. All the experiments presented here were conducted in accordance with the guidelines established by the Hokkaido University Animal Experiment Committees and the ARRIVE guidelines (http://www.nc3rs.org.uk/page.asp?id=1357).

### Fish and creating bone fracture model

Sexually mature adult (5 to 12 months of age) male and female adult zebrafish (*Danio rerio*) and medaka (*Oryzias latipes*) were utilized. In vivo experiments shown in Fig. 1, wild type zebrafish were obtained from RIKEN Brain Science Institute (Saitama, Japan) and wild type medaka were obtained from the Japan National BioResource Project (NBRP) Medaka (Okazaki, Japan). For RNA isolation, both zebrafish and medaka were obtained from a local aquarium store (Homac, Sapporo, Japan). The fish were kept at 28 °C under a 14 h light/10 h dark cycle and automatically fed TetraMin Tropical Flakes (Tetra, Melle, Germany) twice a day. To create the bone fracture model, the fish were anesthetized with tricaine (Sigma–Aldrich, St. Louis, MO, USA) and their tail bones were fractured, using a scalpel, under a microscope. To evaluate the time point that the fracture was completely healed, analysts who did not know the data source were blindly measured it.

### Bone staining

Fishes were sacrificed with an overdose of Tricaine solution (4 g/L in water). The fishes’ caudal fin rays were fixed overnight at 4 °C with 4% paraformaldehyde in PBS for Alizarin red and Alcian blue staining. After fixation, the fin rays were stained with Alcian blue solution (70% ethanol and 30% acetic acid containing 0.1% Alcian blue) at room temperature overnight. The fins were treated with ethanol and washed in water. Alizarin red solution (4% Alizarin red, 0.5% potassium hydroxide) was added and the fins were incubated overnight at room temperature. Stained samples were stored in 80% glycerol for imaging..

### In vivo LIPUS stimulation

One day after the bone fracture, the fish were placed in six-well plates with water (one fish per well). LIPUS (Teijin Pharma, Tokyo, Japan) was generated using an array of six PZT-4 (lead-zirconate titanate) transducers (2.5 cm diameter) fixed with a locking device. The plate containing the fish was placed on the array and the locking device was immersed in a water tank. The LIPUS signal was set to 1.5 MHz, 200 ms burst width sine waves at 1.0 kHz, which were delivered at an intensity of 30 mW/cm^2^. The fish were exposed to LIPUS for 20 min daily for up to four weeks or until the fracture was completely healed. The bone fracture sites were observed using an optical microscope (Nikon, Tokyo, Japan) every day.

### Total RNA extraction from fish fin ray

The fin rays were homogenized using a BioMasher homogenizer (BioMasher II, Nippi, Tokyo, Japan). The homogenate was lysed in TRizol Reagent (Invitrogen, Carlsbad, CA, USA) and total RNA was extracted following the instructions for the RNeasy Mini RNA isolation kit (Qiagen, Hilden, Germany).

### Cell culture and LIPUS stimulation

MLO-Y4 cells were kindly provided by Dr. Lynda F. Bonewald from the Department of Oral Biology at the Kansas City School of Dentistry, University of Missouri, Kansas City, MO, USA. Cells were maintained in α-modified essential medium (α-MEM) containing 10% fetal bovine serum (FBS) at 37 °C in a humidified atmosphere of 95% air and 5% CO_2_. Prior to LIPUS stimulation, the cells were seeded on collagen-coated six-well dishes and precultured overnight. The cell culture plates were loaded onto the LIPUS system array and fixed with the locking device. The locking device was immersed in a water tank and LIPUS stimulation was provided for 20 min (1.5 MHz, pulsed-wave mode intensity of 30 mW/cm^2^). LIPUS system has six ultrasound transducers for use in a six-well cell culture plate. To generate LIPUS treated and non-treated (control) cells in one plate, three ultrasound transducers were plugged and the other three were unplugged to shut down the LIPUS stimulation. The cells were harvested after the LIPUS treatment and total RNA was extracted using a spin column kit (Zymo Research, Orange, CA, USA).

### Quantitative RT-PCR

Total RNA was extracted from the fish fin rays and MLO-Y4 cells. RNA (0.5–1 µg) was reverse transcribed using a high-capacity complementary DNA (cDNA) reverse transcription kit (Applied Biosystems, Forster City, CA, USA). qRT-PCR assays were run and quantified in the ABI STEP one real-time PCR system using SYBR green PCR Master Mix (Qiagen). Relative mRNA expression was determined using the ΔCt method, and the values were normalized to the expression of *β-actin*. The primer sets used for qPCR are shown in Table 3.

### RNA-sequencing and analysis

Total RNA was isolated from the MLO-Y4 cells 30 min after LIPUS stimulation, using a spin column kit (Zymo Research). Total RNA integrity and purity were assessed using an Agilent 2100 Bioanalyzer (Agilent Technologies, Palo Alto, CA, USA). The Illumina TruSeq Stranded mRNA protocol was used for the preparation of RNA-seq libraries and sequenced on a HiSeq 2500 machine (Illumina, San Diego, CA, USA) as paired-end, 100-base pair reads and 20 million reads per sample were obtained. Paired-end sequencing reads from the HiSeq 2500 were trimmed using Trimmomatic v0.36 and trimmed reads were aligned to the GRCm38 genome assembly using TopHat v2.1.0 and bowtie v2.2.6.0. Gene expression intensity was normalized to Fragments per kilobase of exon per million reads mapped (FPKM) which was calculated using Cufflinks (version 2.2.1) with a transcriptome reference (Ensembl Mouse Transcript). Signature genes for each group were identified using an adjusted *q*-value of < 0.05. Significant difference was defined by *q*-value. *q* is an false discovery rate (FDR)-adjusted enrichment *p* value, and *q* < 0.05 (i.e., − log 10(*q*) > 1.3) was defined as significant. A principal component analysis (PCA) was performed using PCAGO, an interactive web service (https://pcago.bioinf.uni-jena.de) to explore the variation between samples. We used a subset of the top 500 genes with most variable expression. The Functional Annotation Tool of the Database for Annotation, Visualization, and Integrated Discovery (DAVID) v6.8 was used to characterize the gene annotation enrichment analysis.

### Target gene analysis of transcription factor

To identify the putative target genes of the transcription factors, the mouse ChIP-seq database in ChIP-Atlas (http://chip-atlas.org/) was used. If a transcription factor’s ChIP-seq peaks settled within the gene’s promoter region (5 kb around the transcription start site), the gene was identified as a target gene^66^.

### Statistics

All statistics were calculated using Microsoft Excel and GraphPad Prism. All data are presented as a mean ± SEM and comparisons were made using Student’s *t*-test. The SEM was chosen to compare the population mean of the two groups. Significance levels of results were defined as follows: * *p* < 0.05, ** *p* < 0.01, and *** *p* < 0.001. All experiments presented were performed in two to three independent experiments, except for the RNA-seq study. The experiments were not randomized and sample size was not predetermined.

## Data Availability

Data supporting the conclusions are available from the corresponding author upon reasonable request. RNA-seq data have been deposited to Gene Expression Omnibus (GEO) repository with accession number GSE162674.
